# Organelle resolved proteomics uncovers PLA2R1 as a novel cell surface marker required for chordoma growth

**DOI:** 10.1186/s40478-024-01751-w

**Published:** 2024-03-07

**Authors:** Shahbaz Khan, Jeffrey A. Zuccato, Vladimir Ignatchenko, Olivia Singh, Meinusha Govindarajan, Matthew Waas, Salvador Mejia-Guerrero, Andrew Gao, Gelareh Zadeh, Thomas Kislinger

**Affiliations:** 1 Princess Margaret Cancer Centre, Princess Margaret Cancer Research Tower, University Health Network, 101 College Street, Room 9-807, Toronto, ON M5G 1L7 Canada; 2 Division of Neurosurgery, Department of Surgery, University of Toronto, Toronto, Canada; 3 Laboratory Medicine Program, University Health Network, Toronto, Canada; 4 Department of Medical Biophysics, University of Toronto, Toronto, Canada

**Keywords:** Organelle fractionation, Proteomics, Cell surface proteins, Chordoma, MAPK signalling

## Abstract

Chordomas are clinically aggressive tumors with a high rate of disease progression despite maximal therapy. Given the limited therapeutic options available, there remains an urgent need for the development of novel therapies to improve clinical outcomes. Cell surface proteins are attractive therapeutic targets yet are challenging to profile with common methods. Four chordoma cell lines were analyzed by quantitative proteomics using a differential ultracentrifugation organellar fractionation approach. A subtractive proteomics strategy was applied to select proteins that are plasma membrane enriched. Systematic data integration prioritized PLA2R1 (secretory phospholipase A2 receptor–PLA2R1) as a chordoma-enriched surface protein. The expression profile of PLA2R1 was validated across chordoma cell lines, patient surgical tissue samples, and normal tissue lysates via immunoblotting*.* PLA2R1 expression was further validated by immunohistochemical analysis in a richly annotated cohort of 25-patient tissues. Immunohistochemistry analysis revealed that elevated expression of PLA2R1 is correlated with poor prognosis. Using siRNA- and CRISPR/Cas9-mediated knockdown of PLA2R1, we demonstrated significant inhibition of 2D, 3D and in vivo chordoma growth. PLA2R1 depletion resulted in cell cycle defects and metabolic rewiring via the MAPK signaling pathway, suggesting that PLA2R1 plays an essential role in chordoma biology. We have characterized the proteome of four chordoma cell lines and uncovered PLA2R1 as a novel cell-surface protein required for chordoma cell survival and association with patient outcome.

**Supplementary Information:**

The online version contains supplementary material available at 10.1186/s40478-024-01751-w.

## Introduction

Chordoma is a rare but devastating bone cancer of the skull base and spine with an incidence of 8 cases per ten million people per year [28]. Chordoma patients experience a relatively high mortality compared to more benign tumor types, with a 10-year survival rate of 40%. Patients also experience morbidities associated with chordoma progression during the course of their disease as 30–85% experience tumor recurrence and invasion into surrounding neurological structures as well as one-third of cases metastasize to other organs [49]. There is growing interest in identifying new targeted therapy options to treat chordomas both upfront after surgery/radiotherapy, and after later tumor recurrence, in order to improve these outcomes. Such novel targeted therapies may be preferentially considered for patients who underwent a subtotal surgical resection or have poorly prognostic molecular signatures, thereby leading to further personalized clinical care [50]. Previous studies using whole genome (WGS) [37], whole exome (WES) [32, 37], and RNA sequencing [4, 32, 37] have shown a high degree of genetic heterogeneity between chordoma tumors and no robust targetable biomarkers have been identified to date. Accordingly, novel approaches to identifying new therapeutic targets are needed and could be transformative for patient care.

Aberrations from homeostatic protein expression, either through somatic mutations or large-scale genomic amplifications/deletions, lead to rewiring of essential signalling pathways that drive cancer initiation and progression [21]. Importantly, these changes are difficult to predict from genomic and transcriptomic data and require direct quantitation at the proteome level, which has not been comprehensively performed in chordoma to date. Mass spectrometry-based proteomic approaches can inform spatial localization of proteins which other omic technologies cannot provide [5]. Cell surface proteins carry out essential cellular functions such as cell–cell interactions, ion transport and cell signalling transduction pathways [3]. Surface proteins are attractive drug targets as they are readily accessible through small molecules, therapeutic antibodies, or other immunotherapies [3].

In this study we used a proteomics-based approach to identify phospholipase A2 receptor (PLA2R1) as a novel chordoma-enriched cell surface protein. We found that PLAR21 expression was significantly associated with poor clinical outcomes in chordoma patients. Knockdown studies revealed that PLA2R1 downregulation led to reduction in 2D, 3D and in vivo chordoma tumor growth. Molecular analyses suggest that PLA2R1 might be a regulator of tumor metabolism in chordoma, possibly through metabolic reprograming via activation of the mitogen-activated protein kinase (MAPK) signalling pathway. Altogether, our work highlights the relevance of PLA2R1 as a candidate for future therapeutic intervention.

## Material and methods

### Ethical statement

All chordoma patient samples in the study were obtained from consenting patients as approved by the University Health Network Brain Tumor Biobank (REB#-18-5820). Animal experiments were performed according to guidelines from the Canadian Council for Animal Care and under protocols approved by the Animal Care Committee (ACC) of the Princess Margaret Cancer Centre (Toronto, ON, Canada), Animal Use Protocol (AUP 6396).

### Cell culture

The chordoma cell lines U-CH17P, U-CH17M, U-CH17S, U-CH11R, UM-Chor1 and MUG-chor1 were generously provided by the Chordoma Foundation (www.chordomafoundation.org). The cells were grown IMDM/RPMI 1640 (4:1; Sarstedt Inc) supplemented with 10% FBS and penicillin–streptomycin-glutamine (PSG; 100 U/mL penicillin, 100 μg/mL streptomycin, 292 μg/mL L-glutamine) (Gibco, Ontario, Canada). The cells were maintained in a humidified 37 °C incubator with 5% CO_2_ until confluency.

### Silica-bead based cell surface protein capture

Cell surface protein capture (in triplicate for each cell line) was performed as previous described [9, 19] with minor modifications. The chordoma cell lines (U-CH17P, U-CH17M, U-CH17S and U-CH11R) were washed three times with ice-cold MES-buffered saline MBS buffer (25 mM MES, 150 mM NaCl pH 6.5) and a monolayer of cells were overlaid with 20 mL of 1% colloidal silica bead (LUDOX® CL colloidal silica suspension in water) with gentle shaking for 15 min at 4 °C. Excessive beads were removed, and the plates were washed three times with MBS buffer. 20 mL of 0.1% Polyacrylic acid (PAA) in MBS buffer was added to the cells with gentle shaking for 15 min at 4 °C. PAA was removed from the plates and fresh sucrose/HEPES buffer (250 mM sucrose, 25 mM HEPES pH 7.4 and 20 mM KCL) was added. Cells were scraped from the plate and centrifuged at 300 xg for 5 min. The supernatant was discarded, and the pellet was added to fresh sucrose/HEPES buffer. Cells were lysed by 3 cycles of sonication (35% amplitude, 10 s). A discontinuous Histodenz density gradient in sucrose/HEPES was prepared at different concentrations (45%, 50%, 55% and 60%) and layered in a 13.2 mL (Beckman) ultracentrifugation tube. The samples were diluted with Histodenz to a final concentration of 20% and added on top of the Histodenz gradient. The samples were centrifuged at 100,000 xg for 2 h in a SW 41Ti rotor (Beckman). After the centrifugation, the density gradient layers were removed, and the resulting pellet was washed with sodium carbonate (pH 12) solution via rotation for 15 min at 4 °C. The beads were centrifuged at 5000 xg for 20 min in a benchtop centrifuge and the supernatant was removed. Elution buffer (400 mM NaCl, 25 mM HEPES, 1% Triton X- 100, pH 7.4) was added to the beads and rotated overnight at 4 °C to elute membrane proteins from the silica beads. The eluted proteins were precipitated with ice cold acetone overnight at − 20 °C. The samples were pelleted at 10,000 xg for 10 min and the resulting pellet was solubilized with 100 µL of lysis buffer I (50% (v/v) 2,2,2,-Trifluoroethanol (TFE) and 50% PBS).

### Subcellular fractionation and protein extraction

Subcellular fractionations (performed in triplicates for each cell lines) were performed as previously described [20] with minor modifications. Chordoma cell lines (U-CH17P, U-CH17M, U-CH17S and U-CH11R) were pelleted and washed three times with PBS. Cells were homogenised in lysis buffer (50 mM Tris–HCL (pH 7.4), 5 mM MgCl_2_, 0.1% Triton X-100 and Protease inhibitors) and kept on ice for 10 min, and further homogenised with a loose-fitting pestle. Sucrose was added to the lysates to a final concentration of 250 mM (isotonic solution). All the subsequent steps were done at 4 °C. The lysates were centrifuged at 800 xg for 15 min in a benchtop centrifuge (Eppendorf 5430R) at 4 °C to separate the nuclear fraction. The resulting supernatant served as the source of cytosol, mitochondria and microsomes (i.e., mixed membranes). The nuclear pellet was further resuspended in 2.5 mL of cushion buffer (2 M sucrose, 50 mM Tris–HCl, 5 mM MgCl_2_, 1 mM dithiothreitol (DTT) and Protease inhibitors – Roche) and overlaid on 2 mL of cushion buffer in ultra-clear open top 5 mL ultracentrifugation tube (Beckman) and centrifuged at 80,000 xg for 45 min (Beckman SW 55Ti rotor). The mitochondrial fraction was isolated from the crude lysate by centrifugation at 8000 xg for 15 min. The resulting pellet was washed with a lysis buffer and spun again to retrieve the mitochondrial fraction. The resulting supernatant was centrifuged at 150,000 xg for 1 h (Beckman SW 55Ti) to isolate the microsomal pellet (mixed membranes). The supernatant served as the cytosolic fraction. Nuclear proteins were extracted from the nuclear fraction with a lysis buffer (20 mM HEPES, 400 mM NaCl, 0.2 mM EDTA) and rotated for 30 min at 4°. The pellet was passed through 18-guage needle several times and centrifuged at 9000 xg for 10 min to isolate the soluble nuclear fraction and insoluble pellet. The resulting organelle pellets (mitochondria, nuclear and microsome) were lysed in 100 µL of lysis buffer (50% (v/v) 2,2,2,-Trifluoroethanol (TFE) and 50% PBS).

### Sample preparation for shotgun proteomics

The pellets obtained from the subcellular fractions were lysed by repeated freeze–thaw cycles (5 cycles, switching between a dry ice/ethanol bath and 60 °C water bath) in lysis buffer. Samples were sonicated on an ultrasonic block sonicator for five 10 s cycles at 10 watts per tube (Hielscher VialTweeter) followed by extraction at 60 °C for 1 h. Disulphide bonds were reduced with 5 mM DTT, followed by 30 min incubation at 60 °C. Free sulfhydryl groups were alkylated by incubating the samples with 25 mM iodoacetamide in the dark for 30 min at room temperature. The samples were diluted (1:5) with 100 mM ammonium bicarbonate (pH 8.0) and 2 mM CaCl_2_ was added. Proteins were digested overnight with 2 µg of trypsin/Lys-C enzyme mix (Promega) at 37 °C. Peptides were desalted by C18-based solid phase extraction, then dried in a SpeedVac vacuum concentrator. Peptides were solubilized in mass spectrometry-grade water with 0.1% formic acid. Liquid chromatography was directly coupled to an Orbitrap Fusion Tribrid (Thermo Scientific) and data was acquired as previously described [24]. Raw files were searched using the MaxQuant software (version 1.5.8.3) against a Uniprot human sequence database (number of sequences 42,041) with an FDR set to 1% for positive peptide spectral matches and protein identification using a target-decoy strategy [20]. Searches were performed with maximum of two missed cleavages, oxidation of methionine residues as a variable modification, and carbamidomethylation of cysteine residues as a fixed modification. Intensity-based absolute quantification (iBAQ) and label-free quantitation (LFQ) were enabled, with match between runs function disabled due to the differences in organellar proteomes. Subsequent analyses were performed using the proteinGroups.txt file. Contaminant sequences and matching decoy were removed, and proteins identified with two or more unique peptides were carried forward. iBAQ intensities were used for protein quantitation. The data was median normalised and missing values were imputed with low values (between 1 and 1.2 log_2_ values).

### Immunohistochemical analysis

A cohort of 25 chordoma patients with formalin-fixed paraffin (FFPE) embedded slides and clinical follow up data were obtained from the University Health Network Brain Tumor Biobank (REB#-18-5820). Slides with 5 µm FFPE tissue sections were rehydrated with serial dilutions of ethanol followed by water and pH 6 sodium citrate dihydrate buffer was used for heat-mediated antigen retrieval. A 3% hydrogen peroxide in methanol solution was utilized to block endogenous peroxidase activity. Blocking solution (5% bovine serum albumin in phosphate buffered saline plus 0.1% Triton X-100) was applied to slides for 1 h at room temperature. Subsequently, primary antibody anti-PLA2R (Millipore Sigma, MABC942, mouse monoclonal antibody) were applied overnight at 4 °C diluted (1:200, 1:200, 1:250, respectively) in blocking solution. A 1 h incubation with secondary antibody was performed followed by processing with the DAKO polymer-HRP system and DAB peroxidase kit, counterstaining with hematoxylin, tissue dehydration, and slide cover slipping. Whole slide digital scanning was performed on all slides and images were analyzed using the HALO Image Analysis Platform (Indica Labs). Each slide was annotated with multiple regions of interest to delineate chordoma tumor tissue. Three independent reviewers assessed slides for all cases (JAZ, OS, and an experienced neuropathologist AG) and representative images were selected. Proportions of stain positive cells were quantified using the HALO software algorithm, defined to identify cells with either membranous or cytoplasmic staining as a fraction of all cells. This algorithm was applied to all annotated tissue sections in an unbiased systematic manner (i.e., all IHC analysis were performed in a blinded manner). Wilcoxon's rank sum test was used to compare values between skull base and spinal chordomas. Univariable and multivariable Cox models were utilized to assess the prognostic utility of stain proportions together with known major prognostic clinical factors (extent of surgical resection and radiotherapy use). For survival analyses results, the upper tertile of PLA2R1 values (samples with higher marker positivity) were compared to the lower two tertiles of values.

### siRNA knockdown and clonogenic assay

The chordoma cell lines (U-CH17M, U-CH17S and UM-Chor1) were seeded in 6-well plates at a density of 2,000 cells per well in DMEM/RPMI media and transfected with three siRNA for PLA2R1 (Cat# SR307882, Origene) and scrambled siRNA (negative control) (Cat# SR30004, Origene) at a concentration of 5 nM using lipofectamine RNAiMax transfection reagent (Invitrogen). After 2 weeks colonies were stained with crystal violet staining (0.5% crystal violet, 25% methanol) and the quantification of the colonies was performed using ImageJ (version).

### PLA2R1 CRISPR/Cas9 knockdown

Two single guide RNA’s (sgRNA) targeting PLA2R1, sg1: CACCGATCACAACCTACTTCTGCAG and sg2: CACCGAGACATAACCTCATTAGCAG and control guide RNA LacZ: CACCGCCCGAATCTCTATCGTGCGG were selected from Toronto KnockOut Library V3 (TKOv3) and obtained from Integrated DNA Technologies (IDT) and were cloned into a lentiCRISPRv2 construct (Addgene) with Cas9 and Puromycin cassette. The constructs were packed into the lentivirus using 2nd generation packaging constructs. Briefly, HEK293T cells were seeded at 500,000 cells in a 6 well plate and grown in DMEM (Dulbecco's Modified Eagle Medium) (Sarstedt Inc) supplemented with 10% FBS and penicillin–streptomycin (0.1 × PS) (Gibco) for 24 h. Next, 500 ng of lentiCRISPRv2 construct (LacZ, PLA2R1 sg1 and sg2), 850 ng of psPAX2 (Addgene), and 353 ng of VSV.G (Addgene) were mixed with Xtreme Gene9 (Roche) at a 1:3 ratio (m:v) in Opti-MEM. After incubation for 30 min at room temperature, the mixture was dropwise transferred to HEK293T cells. Next day, the media was replaced with viral harvesting media (DMEM, 10% FBS, 0.2 g/ml BSA and 0.1 × PS). Viral supernatants were collected 24 h and filtered with a 0.22um filter after transfection. 10 × 10^5^ cells UM-Chor1 cells were plated in 6-well plates containing IMDM/RPMI 1640 supplemented with 10% FBS, penicillin–streptomycin-glutamine (PSG; 100 U/mL penicillin, 100 μg/mL streptomycin, 292 μg/mL L-glutamine) and 8 μg/mL polybrene. Cells were infected with lentivirus containing lentiCRISPRv2 constructs targeting sg1-PLA2R1 (sg1), sg2-PLA2R1 (sg2) and LacZ. The UM-Chor1 cell lines without transduction (NT) were used as a negative control. After 24 h, 3 μg/mL of puromycin was added to the media for 48 h. Knockdown of PLA2R1 was verified by western blotting.

### Spheroid assay

The effect of PLA2R1 downregulation on 3D growth was evaluated by seeding 10 × 10^3^ cells CRISPR/Cas9-edited UM-Chor1 cells (sg1 and sg2) with their respective controls (NT and LacZ) in poly-HEMA-coated round-bottom 96-well plates. Cells were grown at 37 °C in 5% O_2_ for 24 h, and further embedded with 30ul Matrigel (Corning). The spheroids were monitored for 4 weeks, and images were obtained using a 4 × or 10 × objective on a Leica DMi1, equipped with an MC170 HD camera microscope. The quantification of spheroid size was performed using ImageJ (version 1.53 K). The metabolic activity was assessed using alamarBlue fluorometric assay. Briefly, 10ul of alamarBlue reagent was added to the spheroids and incubated at 37 °C for 24 h. The fluorescence was measured at 530 nm excitation and 595 nm emission wavelength using plate reader (Spectramax m5, Molecular devices).

### Seahorse assay

UM-Chor1 negative controls (NT, LacZ) and PLA2R1 KD (sg1 and sg2) cells were plated on 96-well seahorse plate and were grown at 37 °C in 5% O_2_ to reach > 90% confluency. The next day, media was changed to DMEM XF assay media (Agilent) and the plate was allowed to equilibrate for 1 h in the incubator. Cartridges were prepared according to manufactures recommendations. Mitochondrial stress compounds used included Oligomycin (2 μM), FCCP (1 μM; Sigma, C2920) and antimycin A (1 μM). Oxygen consumption rate (OCR) and extracellular acidification rate (ECAR) were measured by XF Seahorse Analyzer (Agilent Technologies).

### Immunoblot

Chordoma cell line pellets and frozen chordoma tissue samples were lysed in RIPA buffer (50 mmol/L Tris pH 7.5, 150 mmol/L NaCl, 2 mmol/L EDTA pH 8.0, 0.5% (v/v) Triton X-100, and Complete protease inhibitor cocktail—Roche, Switzerland). The cells were kept on ice for complete lysis. Cell debris was removed by centrifugation at 16,000 g for 10 min at 4 °C. The protein concentration was determined by BCA assay (Thermo scientific). Commercially available normal tissue lysates from various organs (Brain cortex, cerebellum, skin, stomach, esophagus and spleen) were purchased from Takarabio (USA). Gels were loaded with 10 µg of protein lysates per lane and proteins were separated on 7, 8 or 13% SDS-PAGE gels. The resolved proteins were wet transferred to polyvinylidene fluoride membrane (PVDF) and membranes were incubated in 5% (w/v) milk in Tris-buffered saline Tween-20 (TBST; 10 mmol/L Tris-Base, 150 mmol/L NaCl, 0.05% Tween-20; pH 7.4) for 1 h. After blocking, membranes were incubated with primary antibodies (1:1000 mouse anti-human monoclonal PLA2R1, [Sigma], 1:1000 rabbit anti-human monoclonal Brachyury [Cell Signaling], 1:1000 mouse anti-human monoclonal Lamin B1 [abcam], Cleaved_CASP3 [Cell Signaling], CASP3 [Cell Signaling], cleaved-CASP7 [Cell Signaling], CASP7 [Cell Signaling], cleaved-PARP [Cell Signaling], PARP [Cell Signaling], AKT1 [Cell Signaling], AKT1 pS473 [Cell Signaling], AKT1 pT308 AKT1 pS473, FOX1 [Cell Signaling], FOX1 pT24 [Cell Signaling], GSK-3α [Cell Signaling], GSK-3α pS21 [Cell Signaling], rabbit anti-human monoclonal MEK1/2 [Cell Signaling], rabbit anti-human monoclonal MEK1/2 pS217/221 [Cell Signaling], mouse anti-human polyclonal ERK1/2 [Cell Signaling], mouse anti-human monoclonal ERK1/2 pT202/204 [Cell Signaling], rabbit anti-human monoclonal c-Myc [Cell Signaling], rabbit anti-human monoclonal c-Myc pT58 [Cell Signaling], rabbit anti-human monoclonal c-Myc pS62 [Cell Signaling, and 1:1000 rabbit anti-human monoclonal ß-actin [Novus biologicals]) overnight at 4 °C.

### EdU assay

The EdU cell proliferation assay was performed using Click-IT™ Plus EdU Flow Cytometry Assay Kit (Invitrogen/Thermo Fisher Scientific). Briefly, cells were treated with 10 μM of 5-Ethynyl-2′-deoxyuridine (EdU) for 16 h at 37 °C. Cells were trypsinized and fixed with 4% PFA in PBS. Cells were permeabilized with Click-iT® saponin-based permeabilization reagent, followed by addition of 0.5 mL of Click-iT® reaction cocktail for 30 min according to manufactures instruction. DAPI (4′,6-diamidino-2-phenylindole) 0.5 μg/mL was added for 15 min. Cells were analysed using the BD LSRFortessa™ Cell Analyzer. Cells without EdU staining were used as controls.

### Xenograft model

6–8 week-old male NSG mice (n = 4 per condition) were injected subcutaneously on the right or left flank with 1 × 10^6^ of UM-Chor1 control cells (NT and LacZ) or CRISPR/Cas-9 PLA2R1 edited (sg1 and sg2) cells. Tumor growth was measured weekly using calipers as previously described [22], and tumor volume (*V*) was calculated with the formula *V* = 0.5 × *l* × *w*^2^, where *l* and *w* are the longest and shortest perpendicular measurements, respectively. Tumour growth was monitored for a period of 100 days after which mice were sacrificed with CO_2_ and tumours were resected from connective tissue and weighed.

### Statistical analysis

All the proteomics experiments were performed in triplicates. Applicable data were analyzed and represented using the R statistical environment (v3.6.3). Differential expression analysis was performed using unpaired Welch's *t*-test for statistical analysis, and Benjamini & Hochberg adjusted *p*-value < 0.05 deemed as statistically significant. Visualization in R was performed using the ggplot2 (3.2.1) and ComplexHeatmap (v2.2.2).

## Results

### Subcellular fractionation and plasma membrane enrichment

**Fig. 1:**
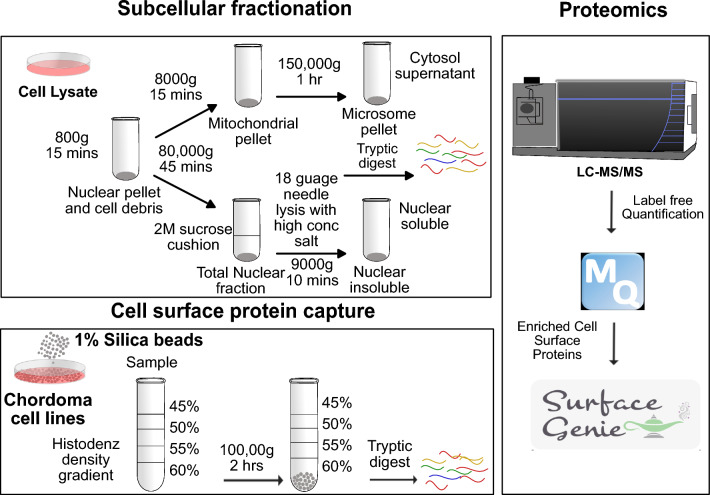
Schematics of silica-bead based surface protein capture and organelle fractionation: Schematic representation of cell surface capture, using the colloidal silica-bead method and organelle fractionation using ultracentrifugation combined with MS-based proteomics

**Fig. 2:**
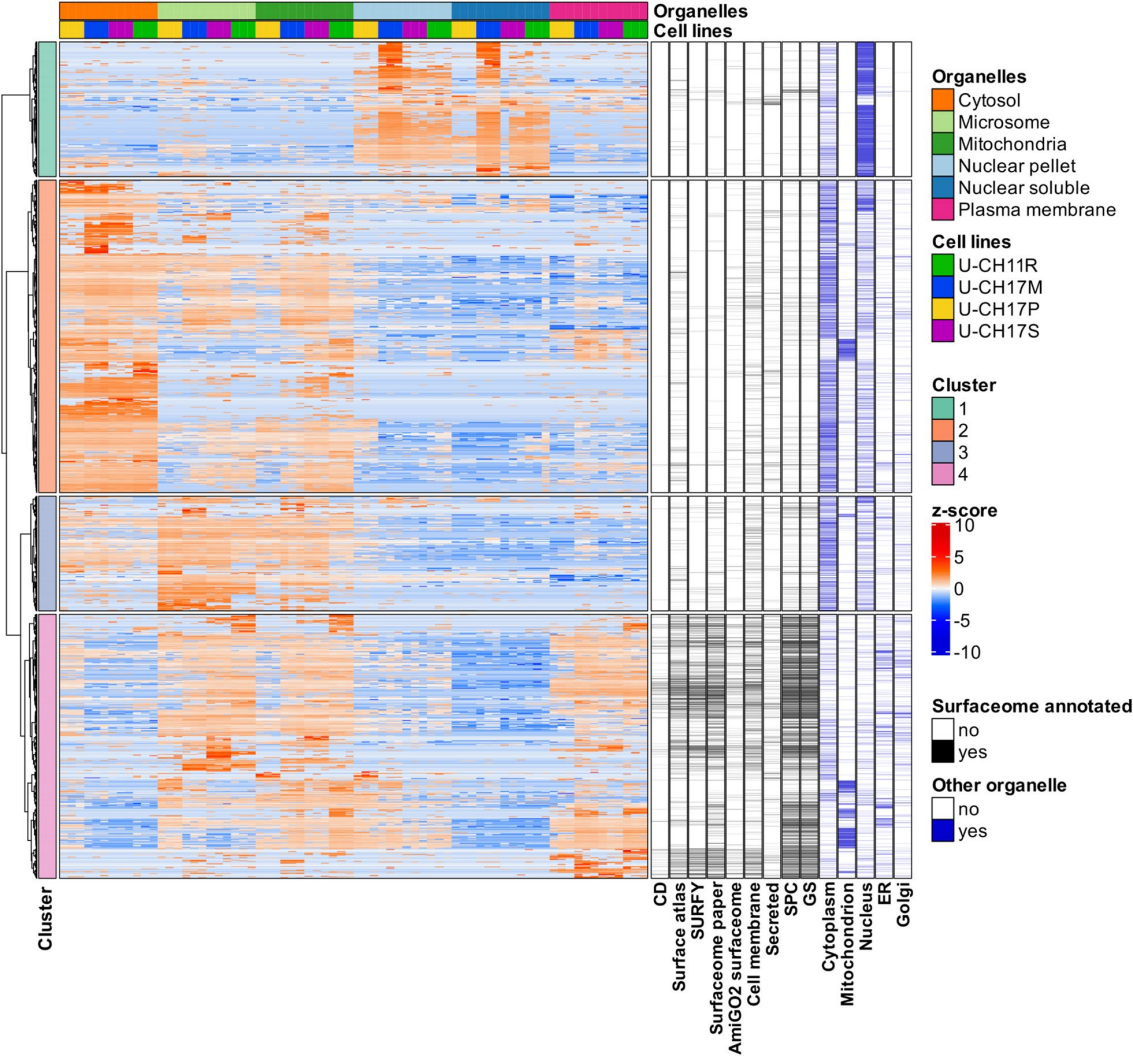
Analysis of sub-cellular proteomes. Heatmap showing four distinct protein clusters using unsupervised hierarchal clustering based on protein abundance (z-score) of all proteins detected. The annotations on the right side (black) indicate surface localization annotation based on several public databases and organelle localization annotation from Uniprot (blue). Cluster annotations  are shown in the annotation bars on the left. The annotation on the top indicates the cell line and organelle type

To explore the surface proteome of chordoma cell lines, we utilized a subtractive proteomics approach [34] that combined silica bead-based plasma membrane capture with organelle fractionation using differential ultracentrifugation (Fig. 1). Organelle fractionation resulted in the enrichment of five subcellular compartments (plasma membrane, cytosol, mitochondria, membrane-derived microsome and two nuclear fractions). In total, 72 liquid chromatography mass spectrometry (LC–MS) analyses were performed, resulting in the detection of 7688 proteins (Additional file 1: Fig. S1a; Additional file 2: Table S1). Principal component analysis (PCA) of the fractions revealed a high degree of similarity between different organelle proteomes regardless of cell line, indicating that the subcellular and surface fractionation methods produce distinct mass spectrometry profiles that correspond to each isolated organelle (Additional file 1: Fig. S1b). Furthermore, unsupervised hierarchical clustering revealed four distinct proteomic clusters separated based on each organelle that coincided with subcellular localization annotations from publicly available databases (Fig. 2). Cluster 1 showed nuclear protein enrichment (Additional file 1: Fig. S1c), while cluster 2 and 3 indicated cytosolic and mixed membrane protein enrichment (Additional file 1: Fig. S1d and e), respectively. Cluster 4 was enriched in proteins with known or predicted cell surface localization (Additional file 1: Fig. S1f).

**Fig. 3:**
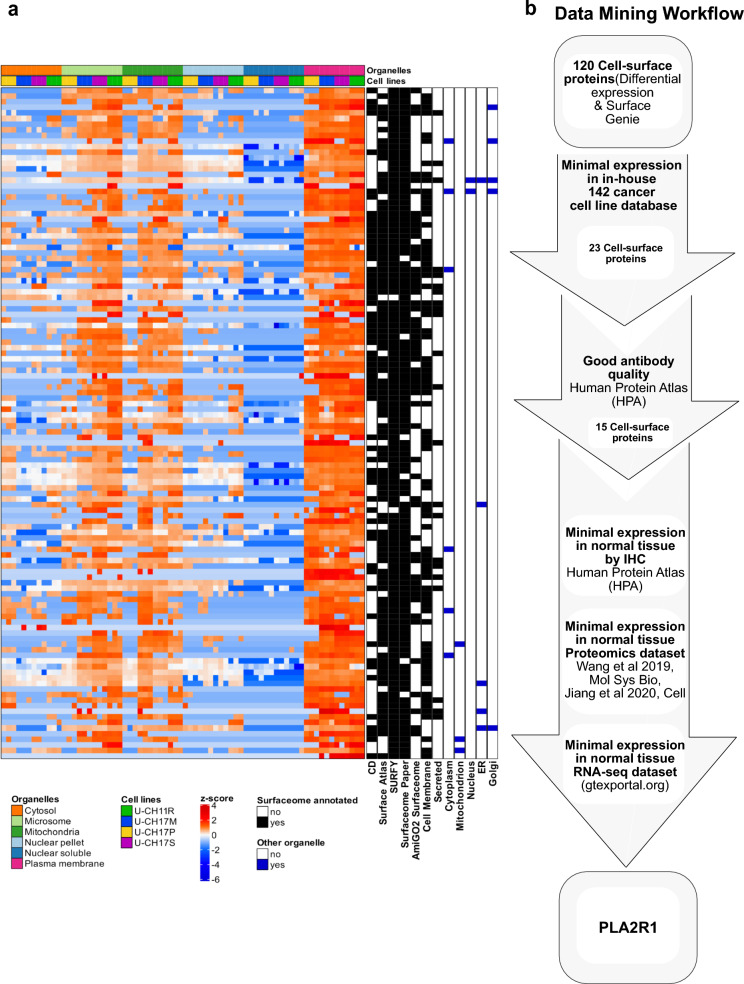
Mining of surface proteins. **a** Heatmap of 120 enriched cell surface proteins. **b** Cell surface protein mining strategy, where the 120 proteins were first filtered for proteins with limited expression in other cancer cell lines and antibody availability. The remaining 15 proteins were then ranked based on normal tissue expression by IHC protein detection [40], median protein expression [18, 44], and median RNA expression in normal tissue datasets. The ranks across all four normal tissue datasets were summed and the highest-ranking protein, PLA2R1, was selected for further evaluation

An integrative data mining strategy was applied to identify high-confidence cell surface proteins for functional evaluation. First, a subtractive proteomics approach that compared protein expression in the silica-bead fraction (plasma membrane) against all other organelles was used to detect differentially abundant proteins (FDR < 0.05, |log_2_FC|> 1, Welch’s *t*-test) separately in each cell line (Additional file 1: Fig. S2a). To further increase our confidence in the surface localization of our candidates, we also leveraged surface prediction annotations through the use of the cell surface protein bioinformatics tool, SurfaceGenie [43] (https://www.cellsurfer.net/surfacegenie; Genie score (GS) ≥ 20 and Surface Prediction Consensus score (SPC) ≥ 3) (Additional file 2: Table S1). Finally, we required surface proteins to be consistently upregulated in the plasma membrane fraction of ≥ 2 chordoma cell lines (Additional file 1: Fig. S2b). Applying these criteria resulted in prioritization of 120 highly enriched cell surface proteins (Fig. 3a).

To identify novel targets with chordoma-enriched expression, we next proceeded to evaluate these 120 high confidence cell surface proteins using public proteomics data of 142 cancer cell lines [11, 14, 26, 45]. This resulted in 23 cell surface proteins with limited expression in these data (≤ 5 cancer cell lines) (Fig. 3b). As we were interested in individually interrogating select targets, we did not further consider proteins that had poor antibody availability as determined by lack of immunohistochemistry (IHC) data or an antibody reliability annotation of “Uncertain” in Human Protein Atlas (HPA). We next assessed the normal tissue expression of the remaining 15 chordoma enriched cell surface proteins using publicly available normal tissue datasets (Fig. 3b and (Additional file 3: Table S2). Specifically, we ranked the set of proteins by the proportion of normal tissue in which they were not detected by IHC in HPA (version 20.1) [40], median protein expression in two normal tissue MS-based proteomics datasets [18, 44], and median RNA expression in the GTEx normal tissue RNA-seq database [27]. Proteins were prioritized based on the rank sum of the four individual normal tissue rankings. The top cell surface protein, secretory phospholipase A2 receptor (PLA2R1) (Fig. 3b; Additional file 1: Fig. S3a–d) was selected for further interrogation.

### Validation of chordoma surface marker PLA2R1

**Fig. 4:**
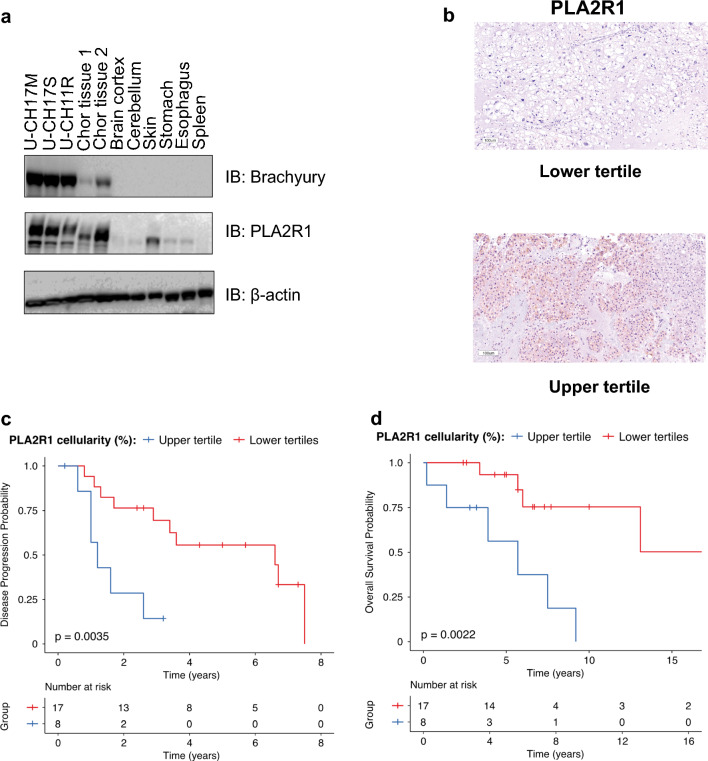
Validation of PLA2R1 in clinical samples. **a** Testing the expression of PLA2R1 in chordoma cell lines (U-CH17M, U-CH17S and U-CH11R), chordoma tissues and commercially available normal tissue lysates by immunoblots. **b** The variability of PLA2R1 expression among tumor samples grouped into an upper tertile and lower two-thirds of expression for visualization. **c** Kaplan Meier plot showing poorer PFI in the top third of patients according to percent of cells positive for PLA2R1 by IHC. **d** Kaplan Meier plot showing that increased PLA2R1% positivity is associated with poorer OS

After prioritizing PLA2R1 as a chordoma-enriched cell surface protein, a commercial antibody was utilized to investigate the expression of PLA2R1 by immunoblotting in chordoma cell lines, chordoma tissue lysates and commercially available normal tissue lysates. As Brachyury is a diagnostic biomarker of chordoma, its expression was used as positive control for the chordoma cell lines and chordoma tissue samples. We were able to validate higher expression of PLA2R1 in the chordoma cell lines and the chordoma tumor tissue samples, compared to normal tissues lysates (Fig. 4a). An additional chordoma cell line (UM-Chor1) and non-chordoma cell line (OVCAR8 – ovarian cancer cell line) as a negative control was also used to test the expression of PLA2R1 (Additional file 1: Fig. S4a). The results indicate that PLA2R1 expression is enriched in chordoma cell lines and tissues, with minimal expression in normal tissue lysates and other cancer cell lines.

### Immunohistochemistry indicates that PLA2R1 expression is associated with poor patient prognosis

We next used IHC of surgically-resected chordoma tissue samples (n = 25) (Additional file 4: Table S3) with the commercially available antibody against PLA2R1 to validate our in vitro chordoma data and assess the utility of PLA2R1 as a clinical biomarker. PLA2R1 expression was observed in patient chordoma tumor samples and localized mainly to cell membranes (Additional file 1: Fig. S4b). Additional file 5: Table S4 shows the potential prognostic utility of PLA2R1 staining proportion, where samples with higher tertile staining values experienced a worse progression-free interval (PFI: HR = 5.2, 95% CI = 1.5–17.5, *p* = 0.0078) and overall survival (OS: HR = 6.7, 95% CI = 1.7–27.0, *p* = 0.0076) in univariable Cox analyses. When combined with extent of resection and adjuvant radiotherapy receipt, which are known prognostic factors, in multivariable Cox analyses, PLA2R1 staining extent was independently associated with poorer clinical outcomes with statistical significance for both PFI (HR = 7.8, 95% CI = 1.8–32.7, *p* = 0.0052) and OS (HR = 11.5, 95% CI = 1.6–80.7, *p* = 0.0144). Representative tumor tissue images of differential PLA2R1 staining proportions are shown in Fig. 4b. Kaplan Meier plots demonstrate the visualization of worse PFI (Fig. 4c) and OS (Fig. 4d) in patients with the upper tertile of PLA2R1 staining percentage.

### Functional interrogation reveals that PLA2R1 is essential for chordoma growth in vitro and in vivo

**Fig. 5:**
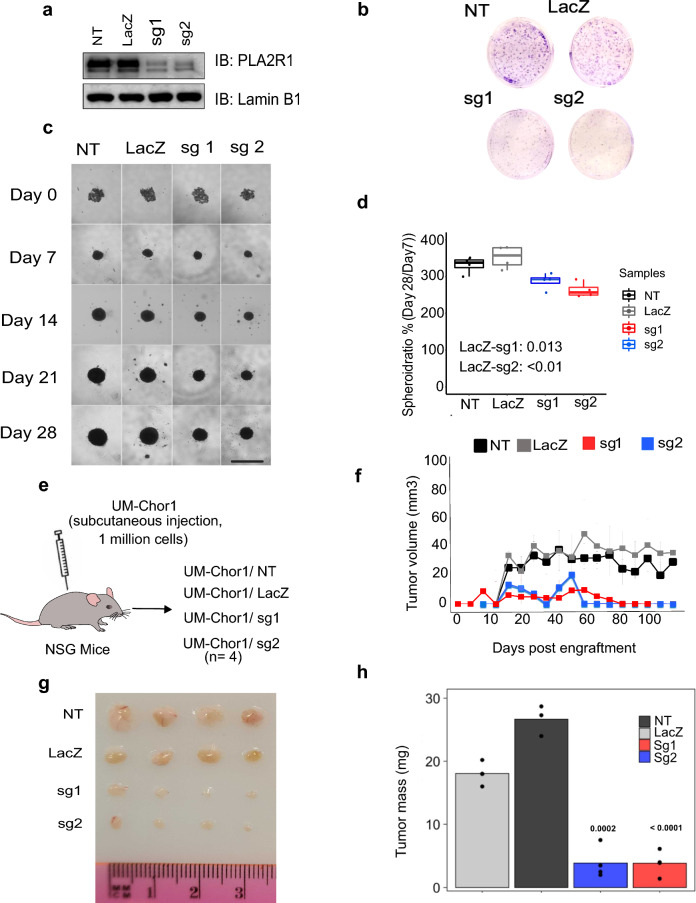
Effect of CRISPR/Cas9 knockdown on chordoma cell growth and survival. **a** Expression of PLA2R1 in UM-Chor1 following CRISPR-mediated knockdown of PLA2R1. **b** Digital image of colony formation assay after PLA2R1 knockdown. **c** Images of spheroid growth showing effect of PLA2R1 KD for a period of 28 days (Scale bar = 500 μM). Boxplots with jitters showing spheroid size (*p*-values calculated with *t*-test test between sg1 and sg2 compared against LacZ). **e** PLA2R1 KD (sg1 and sg2) and controls cells (NT and LacZ) were subcutaneously injected into NSG mice (N = 4). **f** Growth curve of xenograft tumor volume (average values with standard deviation) over a period of 120 days showing PLA2R1 KD negatively impacts in vivo tumor growth compared to controls. **g** Digital image of tumors after sacrifice of mice. **h** Tumor mass (mg) measurement after sacrifice of mice (*p*-values from comparing sg1 and sg2 against LacZ control)

To evaluate the functional role of PLA2R1 in chordoma, we first used siRNA knockdown to assess its essentiality in chordoma cell lines. siRNA knockdown was performed in U-CH17M and U-CH17S and an additional independent chordoma cell line UM-Chor1. The siRNA transfections were performed at 5 nM with 3 different siRNA hairpins for each target, and a pool of all three siRNAs mixed at 1 nM each for a total concentration of 3 nM. After 24 and 48 h of siRNA transfection, immunoblot analysis was performed to assess target protein downregulation in each chordoma cell line. These results revealed reduction of PLA2R1 expression in treated cells compared to control cells (Mock, non-treated and scramble) in all three chordoma cell lines (U-CH17M, U-CH17S and UM-Chor1) after 24 and 48 h (Additional file 1: Fig. S4c). After confirming the knockdown of PLA2R1, colony formation assays were performed. Cells transfected with siRNA targeting PLA2R1 drastically decreased their colony formation ability compared to controls (Additional file 1: Fig. S4d). Next, we evaluated the effect of PLA2R1 downregulation in the UM-Chor1 cell line using CRISPR/Cas9-mediated knockdown. Immunoblot analysis showed downregulation of PLA2R1 with two independent small guide RNAs (sg1 and sg2) when compared to the controls (NT and LacZ) (Fig. 5a). Similar to the transient KD with siRNA, the colony forming ability in PLA2R1 KD cells (sg1 and sg2) were drastically supressed when compared to the negative controls (Fig. 5b), further supporting a role for PLA2R1 in chordoma cell survival and proliferation. To evaluate the impact of PLA2R1 KD on 3D cell growth, we grew the PLA2R1 KD and control cells in low adhesion plates for a period of 28 days (Fig. 5c) to induce the formation of spheroids. The KD spheroids showed significant decrease (*p* < 0.0001) in size when compared to control spheroids (LacZ) (Fig. 5d). At experimental endpoint (28 days), metabolic viability of the spheroids was measured by alamarBlue fluorometric assay. The assay indicated that cellular metabolic activity of chordoma spheroids was reduced by PLA2R1 KD, suggesting PLA2R1 might play a role in metabolic reprogramming leading to reduced growth and survival (Additional file 1: Fig. S5a).

To assess the impact of PLA2R1 KD in vivo*,* we subcutaneously injected 1 million CRISPR/Cas9 mediated PLA2R1 KD (sg1 and sg2) cells and their respective controls (NT and LacZ) into NSG mice (Fig. 5e). Tumor volumes were measured biweekly for a period of 120 days. The mice injected with PLA2R1 KD cells (sg1 and sg2) had significant decrease in tumor size (Fig. 5f) and tumor mass (*p*-value 0.0002 and 0.0001) (Fig. 5g–h) when compared to control (LacZ). These results indicate that PLA2R1 downregulation compromises chordoma cell proliferation and reduces tumor growth in vivo*.*

### PLA2R1 downregulation causes reduction in metabolic activity via MAPK pathway

Cell proliferation was assayed by plating 5 × 10^5^ cells (NT, LacZ, sg1 and sg2) in 6-well plate. The cells were counted for a period of 16 days, PLA2R1 KD showed reduced cell proliferation compared to the control (LacZ) (Additional file 1: Fig. S6a). In parallel, we performed an EdU based cell proliferation assay to examine the effects of PLA2R1 KD on cell proliferation. The results showed an increase of PLA2R1 KD (sg1 and sg2) cells in the G1 phase (*p* < 0.0001) and a significant decrease in S-phase of cell cycle compared to LacZ control (Additional file 1: Fig. S6b). These results suggest that PLA2R1 KD leads to a partial cell cycle arrest in G1 phase in the KD cells, which might result in supressed proliferation and growth (Additional file 1: Fig. S6c–f).

**Fig. 6:**
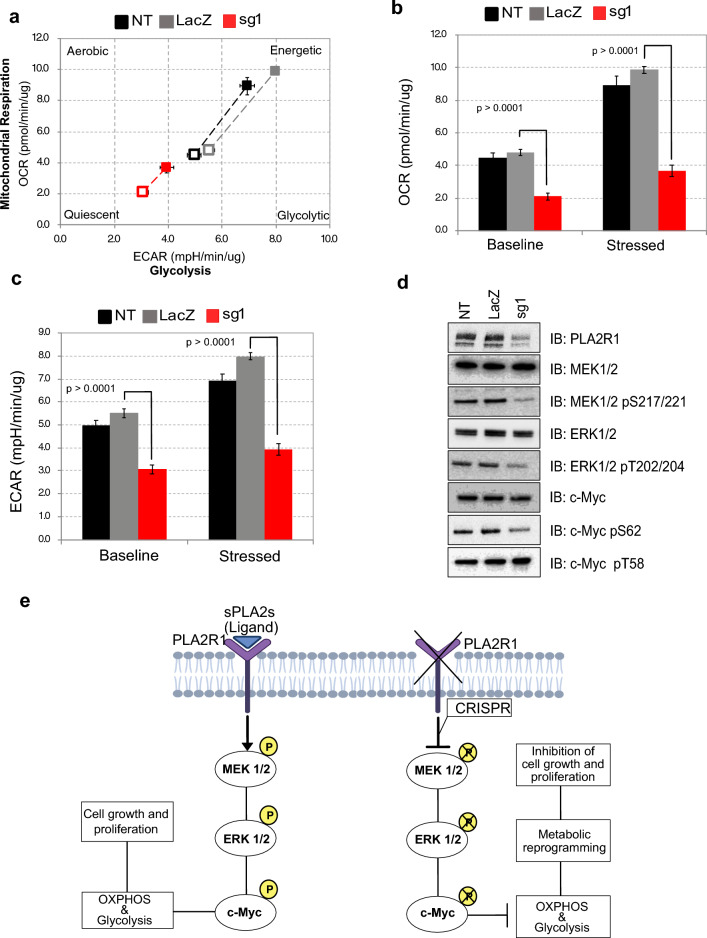
Effect of PLA2R1 knockdown causes metabolic reprogramming via MAPK pathway. **a** Graph illustrating the Seahorse Cell Energy Phenotype of PLA2R1 KD cells and controls (NT and LacZ) and depicting alterations in OCR and ECAR levels both prior to (baseline) and following (maximal) the introduction of mitochondrial stressors. **b** and **c** Barplot showing differences in baseline and maximal OCR and ECAR (Error bars with SD and *p*-values *t*-test comparison between sg1 compared against LacZ). **d** Immunoblots for phosphorylated and total MEK1/2, ERK1/2 and c-Myc post PLA2R1 downregulation. **e** Proposed model showing PLA2R1 is involved in MAPK/ERK signalling pathway. PLA2R1 knockdown in chordoma cells causes metabolic reprogramming by reducing OXPHOS and glycolysis, hence inhibiting cell growth and proliferation mediated by the MAPK pathway

To further interrogate changes in cellular metabolism following PLA2R1 KD, as suggested by reduced fluorescence the alamarBlue assay, we next examined the OXPHOS capacity of PLA2R1 KD UM-Chor1 cells compared to the CRISPR controls. We conducted a standard mitochondrial stress test, where the rate of oxygen consumption (OCR) is measured to assess mitochondrial respiration and the extracellular acidification rate (ECAR) measured to assess glycolytic capacity. At the initial respiration level, CRISPR control cells (NT and LacZ) exhibited elevated OCR and ECAR levels compared to the KD cells (Fig. 6a–c). Upon introducing stressors, which amplifies OCR and ECAR, control cell lines displayed a more significant increase in OCR and ECAR levels compared to KD cells (Fig. 6a–c). This observation suggests a lower oxidative phosphorylation (OXPHOS) and glycolytic capacity in the PLA2R1 KD cell lines, hence indicating reduced metabolic activity due to PLA2R1 downregulation.

Since knockdown of PLA2R1 compromised chordoma cell growth (observed by the colony formation and spheroid formation assays) and reduced metabolic activity (observed by the seahorse and alamarBlue assays), we investigated the expression of apoptotic markers in PLA2R1 KD cells (sg1 and sg2) compared to controls (NT and LacZ) by immunoblotting. The apoptotic markers, cleaved Caspase-3, cleaved Caspase-7 and cleaved PARP were not detected in KD or control cells by immunoblotting (Additional file 1: Fig. S5b–c), indicating that PLA2R1 downregulation does not activate caspase-based cell death pathways. This suggests that the cellular growth defect of PLA2R1 KD is mediated via different mechanisms. We next investigated whether PLA2R1 downregulation alters the PI3K/AKT survival pathway. The phosphorylation of AKT and downstream proteins were unchanged with downregulation of PLA2R1 as observed by immunoblot analysis (Additional file 1: Fig. S5d). Since our CRISPR/Cas9 functional experiments suggested reduced metabolic activity due to PLA2R1 downregulation, it led us to explore the involvement of MAPK/ERK pathway. We performed immunoblotting against phosphorylated MEK1/2 and downstream targets ERK1/2 and c-Myc following PLA2R1 KD. Knockdown of PLA2R1 decreased the levels of phosphorylated MEK1/2 (Ser217/221), ERK1/2 (Thr202/204) and downstream substrate c-Myc at Ser62 position, which is phosphorylated by ERK1/2 (compared to the controls NT and LacZ) (Fig. 6d). The phosphorylation of c-Myc at Thr58, which is a site phosphorylated by GSK3β via a different pathway, was unchanged compared to controls. These findings indicate PLA2R1 KD results in metabolic reprogramming potentially mediated by the MAPK signaling pathway, ultimately leading to reduced chordoma cell proliferation and growth (Fig. 6e).

## Discussion

Chordomas are rare bone cancers of the skull base and spine originating from notochordal remnants [2, 28, 33]. Despite comprehensive surgical resection and adjuvant radiation therapy, local recurrence and metastasis occur in chordoma patients which leads to devastating neurological morbidities and mortality [47]. Currently there are no FDA approved drugs for treating chordomas [38, 39], and the lack of therapeutic interventions has led to a growing interest in understanding the proteome of chordomas to find potential therapeutic targets in order to prevent or treat disease progression and improve clinical outcomes.

Cell surface proteins are attractive therapeutic targets yet are challenging to profile with typical proteomic workflows. Several cell surface protein enrichment techniques such as the cationic colloidal silica-bead method [9], cell surface capture (CSC) [46] and *N-*glycopeptide capture [10] have been established. These technologies have benefits and shortcomings, depending on the type of biological sample and starting material [21]. In this project, we have used a cationic colloidal silica-bead method coupled with organelle fractionation to perform deep proteomic analyses of chordoma cell lines. These comprehensive data were mined with the goal of discovering novel chordoma cell surface markers. We utilized subtractive proteomics to filter plasma membrane proteins against all the other organelle fractions. This proteomic approach is an efficient way to identify components/proteins of one cellular organelle from other cofractionating organelles or fractions [34]. To further increase our confidence in the surface localization of our candidates, we leveraged the cell surface protein bioinformatics tool SurfaceGenie [43]. This resulted in 120 chordoma associated cell surface proteins for further evaluation.

As there are no cell line models mimicking the normal state of the notochord, it is difficult to experimentally characterize cancer associated proteins compared to the normal state (notochord). Instead, several publicly available normal tissue datasets were used to prioritize chordoma enriched cell-surface proteins. Specifically, we used antibody-based staining from the Human Protein Atlas [40], two comprehensive MS-based proteomics datasets of normal human tissues [18, 44] and normal tissue transcriptional profiles from the GTEx portal [27] to evaluate the expression of chordoma cell surface proteins at the protein and transcript level in normal tissues. The goal was to prioritize chordoma specific cell surface proteins, with minimal expression in normal tissues. This systematic evaluation is important for developing targeted therapies, in order to minimize potential toxicities in normal tissue where expression of these targets may be at high levels [23, 36]. This resulted in prioritization of PLA2R1, as a putative target. The expression of PLA2R1 was also confirmed in our chordoma patient cohort using IHC staining. The IHC analysis showed PLA2R1 expression to be localised to cell membranes and present within majority of chordoma patient tissue samples to various degrees.

Brachyury is a well-known biomarker for chordoma [29, 42] that is present in all samples and utilized for neuropathological diagnosis. Unfortunately, brachyury has no clinical utility for patient prognostication and there are limited prognostic indicators that exist for chordoma. In this study, we observed that chordoma patients with elevated PLA2R1 expression had significantly worse clinical outcomes including overall survival and disease-free interval, suggesting that PLA2R1 may have prognostic value for chordoma. Further validation in large, independent cohorts will be required to fully elucidate the prognostic utility of PLA2R1 in chordoma. Biochemically, PLA2R1 is a type I transmembrane receptor, belonging to the mannose receptor family that binds several secreted phospholipases A2 (sPLA2s), collagen and carbohydrates [17, 41]. Despite a substantial degree of sequence similarity, each member of the mannose receptor family exhibits distinct target binding preferences [12, 31], suggesting minimal biological redundancy. The detailed role of PLA2R1 in cancer is yet to be determined, but several studies have shown that PLA2R1 has a tumor suppressive role in several other cancers [1, 41] and has aberrant expression in prostate cancer [13]. To assess the role of PLA2R1 in chordoma, we downregulated protein expression via siRNA and CRISPR/Cas9. We found a significant reduction in cell growth and proliferation in all tested chordoma cell lines, suggesting that PLA2R1 plays an essential role in chordoma cell growth. Interrogation of publicly available genome-wide CRISPR/Cas9 screening data (www.depmap.org/portal) revealed that PLA2R1 was not an essential gene in any other cancer type, suggesting that PLA2R1 demonstrates a chordoma specific vulnerability. This is also in line with the limited detectable protein expression in a panel of 142 cancer cell line proteomes [11, 14, 26, 45]. Assessing the metabolic viability of chordoma cell line-derived spheroids using the alamarBlue fluorometric assay, revealed a decrease in cellular metabolic activity following PLA2R1 knockdown, indicating a potential involvement in metabolic reprogramming. Subsequently, we moved on to investigate how the downregulation of PLA2R1 impacted the in vivo growth of chordoma cells using xenograft models. The in vivo chordoma growth was substantially reduced with PLA2R1 KD (sg1 and sg2) when compared to controls. Furthermore, a flow cytometry EdU assay demonstrated that PLA2R1 downregulation negatively impacts cell cycle progression, suggesting cell proliferation and growth is supressed by PLA2R1 knockdown, further confirming the essentiality of PLA2R1 in chordoma.

Metabolic reprogramming is one of the hallmarks of cancer [15], as metabolic pathway fluxes are required to fulfill heightened energy and synthesis needs of cancer cells [7]. To further explore observed changes in cellular metabolism (as indicated by reduced fluorescence in the alamarBlue assay) following PLA2R1 KD, we performed a seahorse assay, which demonstrated reduction in both OCR and ECAR levels, suggesting PLA2R1 knockdown affects both glycolytic and OXPHOS metabolism. The majority of malignancies depend on glycolysis, which contributes to approximately 60% of their total ATP production [8], however OXPHOS respiration is still activated and substantially contributes for energy requirements in cancer cells [6]. The reduction of metabolic activity upon PLA2R1 KD led us to investigate the MAPK signalling pathway, which has been reported to be involved in cellular metabolism and proliferation [25, 30]. Indeed, upon KD of PLA2R1, we observed decrease in phosphorylation of MEK1/2, ERK1/2 and c-Myc specifically at the Ser62 site but not at Thr58. The c-Myc Ser62 site is directly phosphorylated by ERK to stabilize c-Myc [35] whereas the phosphorylation of c-Myc at Thr58 has been reported to be phosphorylated by GSK3β via a different pathway which leads to proteasomal degradation [16, 48]. Interestingly, activation of apoptotic markers and downregulation of the PI3K/AKT pathway in PLA2R1 KD cells were not observed, suggesting that our observed phenotypes are mediated through the MAPK/ERK pathway. Though our work represents the first mechanistic investigation of PLA2R1 in chordoma, future work profiling the molecular features of PLA2R1 loss-of-function models can be used to provide insights regarding additional signaling pathways mediated by PLA2R1 in chordoma.

In conclusion, we investigated the proteome of four chordoma cell lines, revealing several novel cell surface protein candidates. The protein expression of PLA2R1 was confirmed through IHC staining in a cohort of richly annotated chordoma patient samples. Our results demonstrated that higher PLA2R1 expression is associated with poor prognosis in chordomas. Utilizing loss-of-function experiments (siRNA and CRISPR/Cas9), we demonstrated that knockdown of PLA2R1 decreased cell growth and proliferation in vitro and in vivo. Mechanistically our data suggests that observed growth defects following PLA2R1 KD are integrated via metabolic reprogramming through the MAPK signalling pathway, but more detailed studies are required in the future to pinpoint the precise molecular mechanism and to translate PLA2R1 as a therapeutic target in chordoma.

### Supplementary Information

**Additional file 1. Fig. S1**: Protein detection and subtractive proteomics. a) Bar plot showing the number of proteins detected in each organelle fraction with the dots on the bar representing number of replicates. b) Principal component analysis (PCA) showing proteomic separation among the organelle fractions. c-f) Bar plot showing the number of proteins detected in the four clusters (through hierarchal clustering) corresponding to organelle selective proteins from different databases. **Fig. S2**: Differential expression analysis between organelle fractions. a) Venn diagram showing overlap between the plasma membrane fraction with all the other organelle fractions in the four chordoma cell lines. Proteins significantly enriched in plasma membrane fractions with a Geniescore of >20 and SPC score of 3 and the following cut-off values (FDR 1) are indicated in red. b) Upset plot showing the overlap of differentially expressed proteins with Genie score and SPC cut-off in the four chordoma cell lines. **Fig. S3**: Expression of PLA2R1 in HPA and publicly available proteomics and RNA-seq datasets. a) IHC staining of PLA2R1 in normal tissue as reported by HPA. 1 = Low expression; 2 = medium expression; 3 = high expression. b) Bar plot shows log2 iBAQ intensities of PLA2R1 in different normal tissues in the Wang et al. [44] proteomics dataset. c) Boxplots show log2 normalised protein abundance of PLA2R1 in the GTEx proteomics dataset, with bar plot showing number of samples the protein was detected in. D) Boxplots show log2 TPM+1 of PLA2R1 in GTEx transcriptomics dataset, with bar plot showing number of samples the protein was detected in different normal tissues. **Fig. S4**: Validation of PLA2R1. a) Immunoblots showing expression of PLA2R1 in an additional chordoma cell line, UM-Chor1 and in a negative control ovarian cancer cell line, Ovcar8. b) Representative IHC image showing expression of PLA2R1 localized to cell membrane. c) Expression of PLA2R1 in U-CH17M, U-CH17S and UM-Chor1 cell line after 24 and 48 hours of siRNA knockdown. d) Digital images of colony formation assay after siRNA KD of PLA2R1. **Fig. S5**: a) Boxplot with jitters showing almarBlue fluorometric intensity of the spheroids (p-values calculated with t-test, between sg1 and sg2 compared against LacZ). b) Immunoblots showing expression of total PARP and caspases in PLA2R1 KD cells and controls. c) Immunoblots showing that cleaved PARP, Caspase 3 and Caspase 7 expression are not observed after PLA2R1 KD. d) Immunoblots for total and phosphorylated AKT1 and its downstream substrate. The immunoblots show no change in phosphorylation of AKT and its downstream substrates. **Fig. S6**: Effect of PLA2R1 knockdown on cell proliferation. a) Plot showing cell proliferation by cell counting for a period of 16 days. The PLA2R1 KD cells show a slow down in growth compared to controls. b) Stacked barplot from EdU cell proliferation assay (n = 3) showing percentage (mean values from three experiments) of cells in different phases of cell cycle G0-G1 phase, S-phase and G2-M phase of PLA2R1 KD cells (sg1 and sg2) and controls (NT and LacZ). There is a higher percentage of cells in G0-G1 phase in PLA2R1 KD cells compared to control (LacZ) (p- values calculated with t-test comparison between sg1 and sg2 compared against LacZ). Lower percentage of cells in S-phase of cell cycle (p-values calculated with t-test comparison  between sg1 and sg2 compared against LacZ). c–f) Representative images of EdU flow cytometry experiment of PLA2R1 KD cells (sg1 and sg2) and control cells (NT and LacZ).

**Additional file 2. Table S1**: Proteins detected by mass spectrometry and differential expression.

**Additional file 3. Table S2**: Top ranked chordoma candidate markers.

**Additional file 4. Table S3**: Clinical characteristics of IHC patient cohort.

**Additional file 5. Table S4**: Univariable and multivariable Cox analysis of IHC data.

## Data Availability

All mass spectrometry raw data has been deposited to the Mass Spectrometry Interactive Virtual Environment (MassIVE) with the following accession MSV000089171 at ftp://MSV000089171@massive.ucsd.edu.
