# Supplementary material for: Organelle resolved proteomics uncovers PLA2R1 as a novel cell surface marker required for chordoma growth
Source: Acta Neuropathol Commun. 2024 Mar 7;12:39. doi: 10.1186/s40478-024-01751-w (PMC10921702; doi:10.1186/s40478-024-01751-w)

Organelle resolved proteomics uncovers PLA2R1 as a novel cell surface marker required for chordoma growth

Shahbaz Khan, Jeffrey Zuccato, Vladimir Ignatchenko, Olivia Singh, Meinusha Govindarajan, Matthew Waas, Salvador Mejia-Guerrero, Andrew Gao, Gelareh Zadeh & Thomas Kislinger

Corresponding author: thomas.kislinger@utoronto.ca (T. Kislinger)

Supplementary figure legends

Supplementary Fig. S1: Protein detection and subtractive proteomics. a) Bar plot showing the number of proteins detected in each organelle fraction with the dots on the bar representing number of replicates. b) Principal component analysis (PCA) showing proteomic separation among the organelle fractions c-f) Bar plot shows the number of proteins detected in the four clusters (through hierarchal clustering) corresponding to organelle selective proteins from different databases.

Supplementary Fig. S2: Differential expression analysis between organelle fractions. a) Venn diagram showing overlap between the plasma membrane fraction with all the other organelle fractions in the four chordoma cell lines. Proteins significantly enriched in Plasma membrane fractions with a Geniescore of >20 and SPC score of 3 and the following cut-off values (FDR < 0.05; log2 fold change > 1) are indicated in red. b) Upset plot showing the overlap of differentially expressed proteins with Genie score and SPC cut-off in the four chordoma cell lines.

Supplementary Fig. S3: Expression of PLA2R1 in HPA and publicly available proteomics and RNA-seq datasets. a) IHC staining of PLA2R1 in normal tissue as reported by HPA. 1 = Low expression; 2= medium expression; 3 = high expression. b) Bar plot shows log2 iBAQ intensities of PLA2R1 in different normal tissues in the proteomics dataset. c) Boxplots show log2 normalised protein abundance of PLA2R1 in the GTEx proteomics dataset, with bar plot showing number of samples the protein was detected in different normal tissues. ID) Boxplots show log2 TPM+1 of PLA2R1 in GTEx transcriptomics dataset, with bar plot showing number of samples the protein was detected in different normal tissues.

Supplementary Fig. S4: Validation of PLA2R1**.** a) Immunoblots showing expression of PLA2R1

in an additional chordoma cell line, UM-Chor1 and in ovarian cancer cell line, Ovcar8. b) Representative IHC image showing expression of PLA2R1 localized to cell membrane. c) Expression of PLA2R1 in U-CH17M, U-CH17S and UM-Chor1 cell line after 24 and 48 hours of siRNA knockdown. d) Digital images of colony formation assay after siRNA KD of PLA2R1.

Supplementary Fig. S5: a) Boxplot with jitters showing almarBlue fluorometric intensity of the spheroids (p-values calculated with t-test, between sg1 and sg2 compared against LacZ). b) mmunoblots showing expression of total PARP, Caspase in PLA2R1 KD cells and controls 3. c) Immunoblots for cleaved PARP, Caspase 3 and Caspase 7 are not observed after PLA2R1 KD. d) Immunoblots for total and phosphorylated AKT1 and its downstream substrate. The immunoblots show no change in phosphorylation of AKT and its downstream substrates.

Supplementary Fig. S6: Effect of PLA2R1 knockdown on cell proliferation. a) Plot showing cell proliferation by cell counting for a period of 16 days. The PLA2R1 KD cells show slowdown in growth compared to controls. b) Stacked Barplot from EdU cell proliferation assay (n=3) showing percentage (mean values from three experiments) of cells in different phases of cell cycle G0-G1 phase, S-phase and G2-M phase of PLA2R1 KD cells (sg1 and sg2) and controls (NT and LacZ). Higher percentage of cells in G0-G1 phase in PLA2R1 KD cells compared to control (LacZ) (p-values calculated with t-test comparison test between sg1 and sg2 compared against LacZ). Lower percentage of cell in S-phase of cell cyle (p-values calculated with t-test comparison test between sg1 and sg2 compared against LacZ). c-f) Representative images of EdU flow cytometry experiment of PLA2R1 KD cells (sg1 and sg2) and control cells (NT and LacZ).

Supplementary Table S1: Proteins Detected by Mass Spectrometry and differential expression analysis

Supplementary Table S2**:** Univariable and multivariable Cox analyses of IHC data

Supplementary Fig. S1

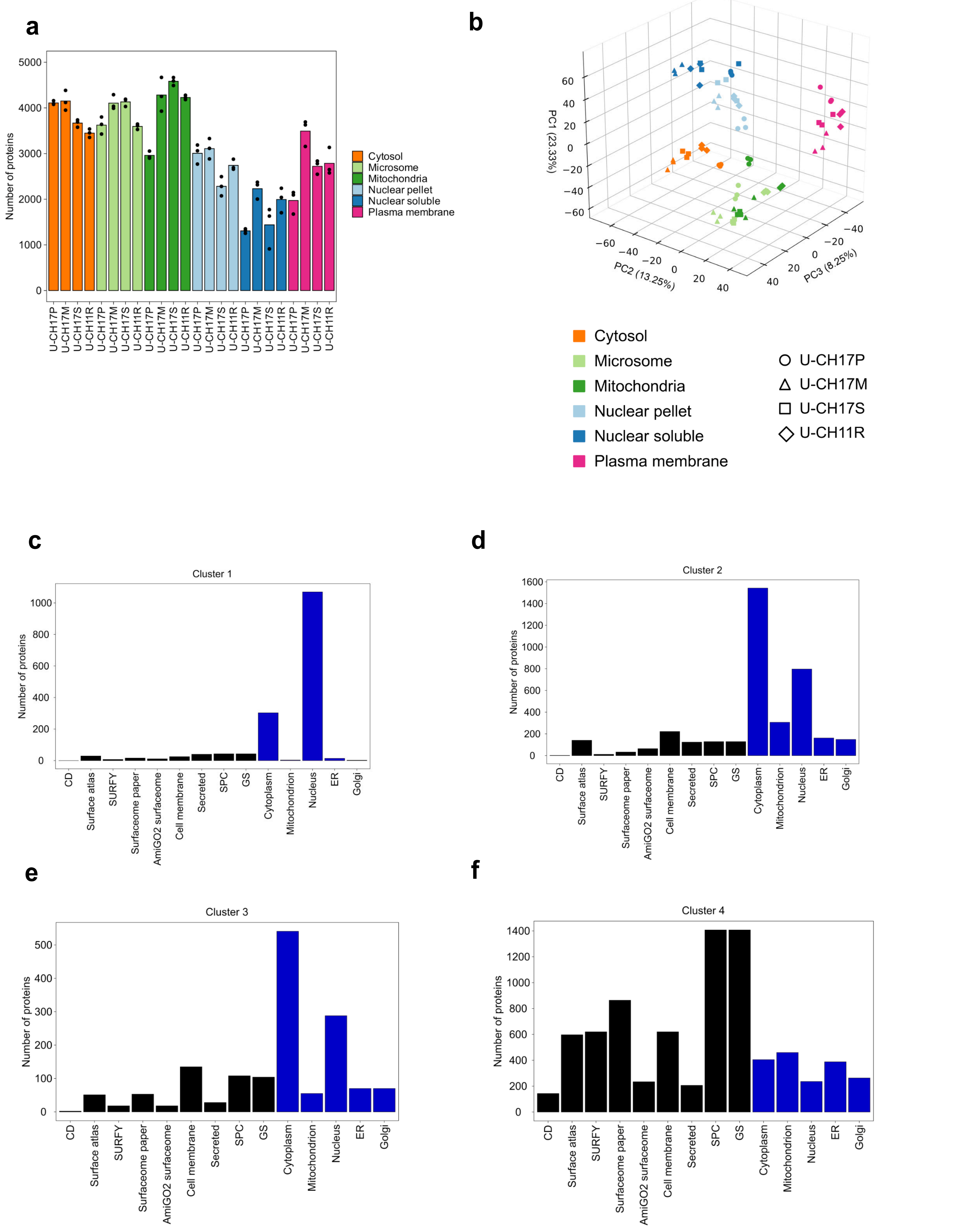

Supplementary Fig. S2

a

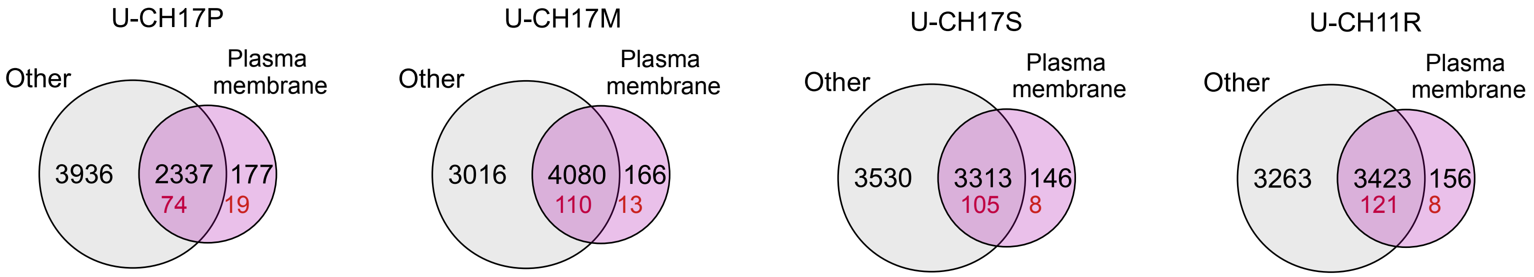

b

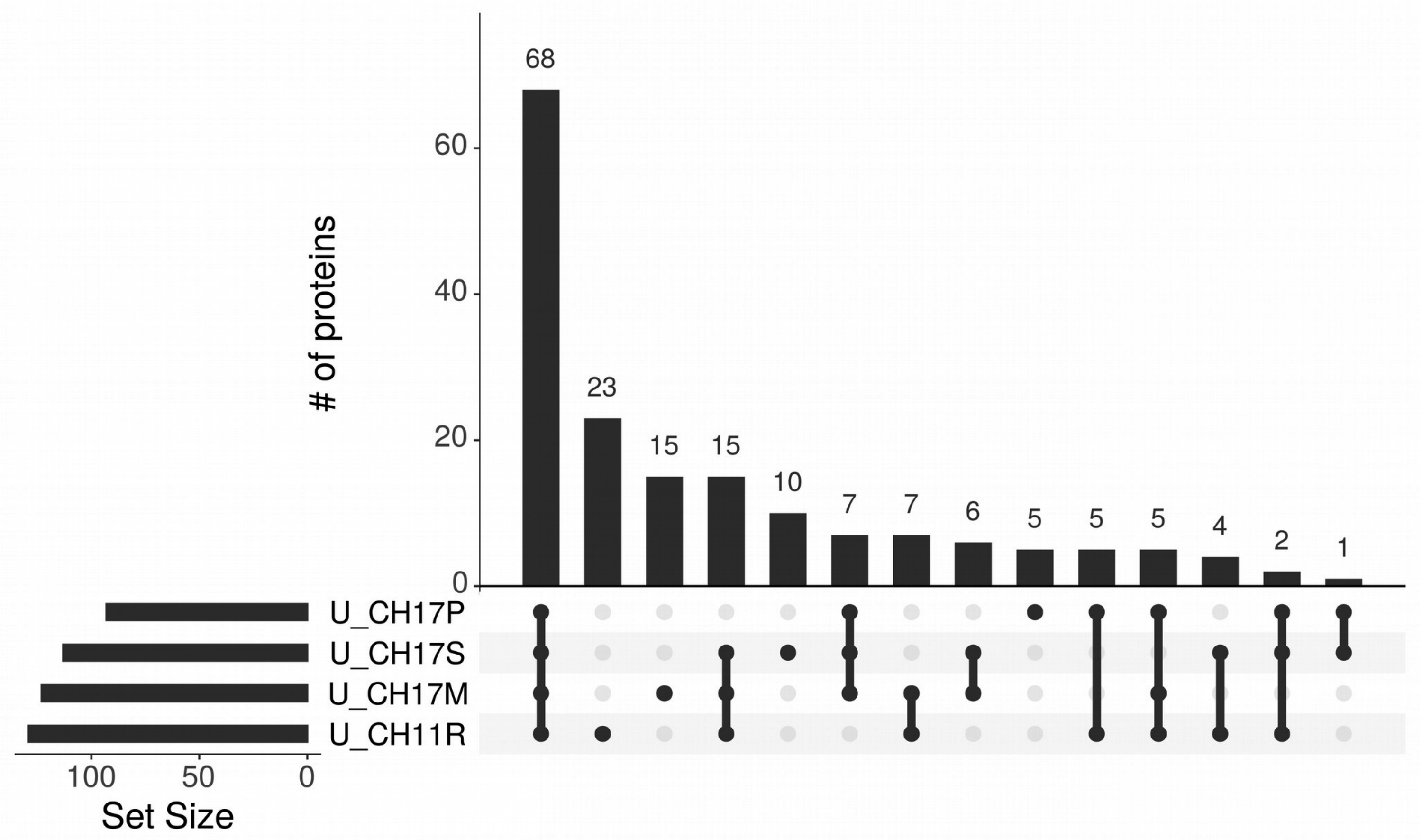

Supplementary Fig. S3

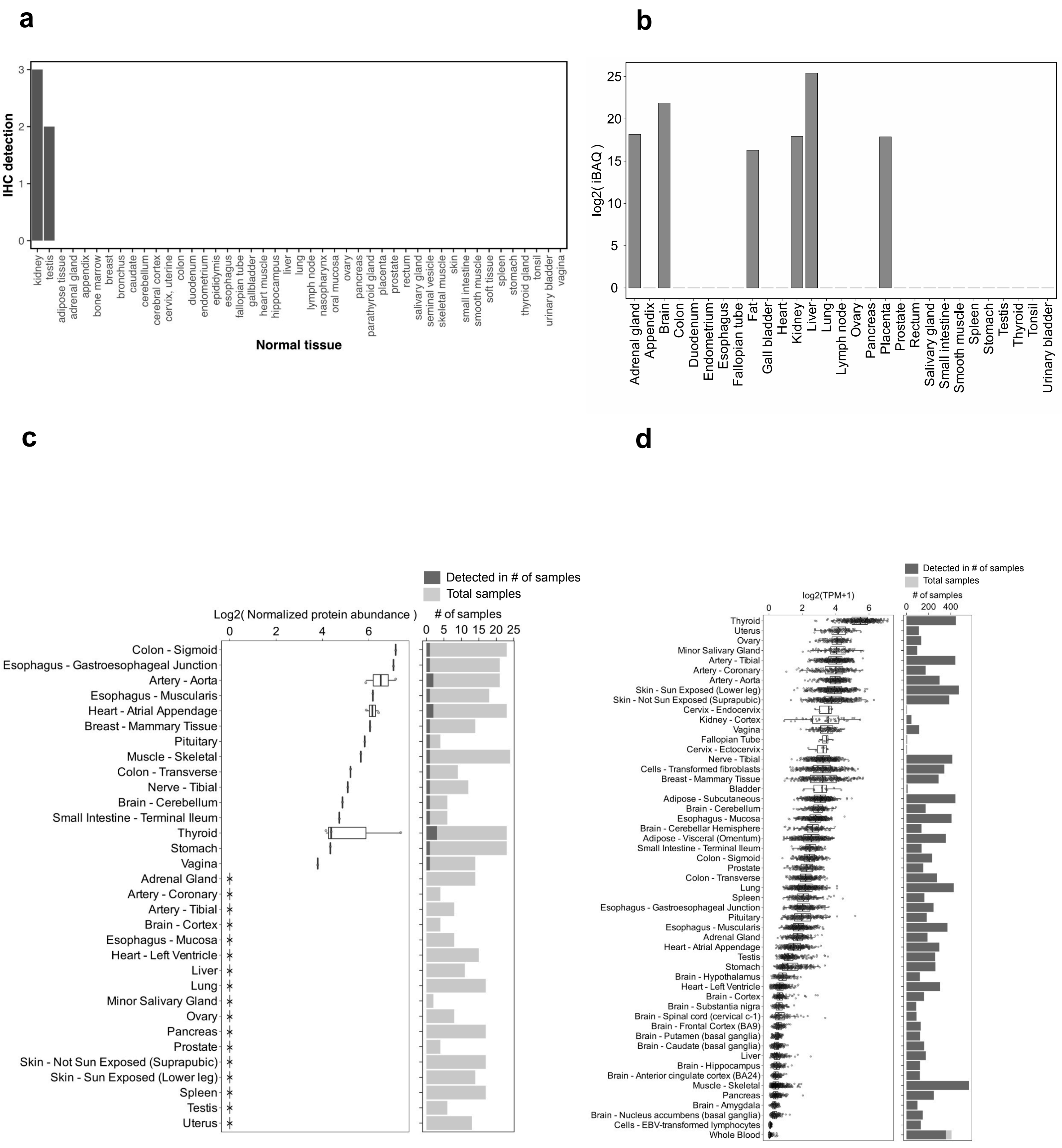

Supplementary Fig. S4

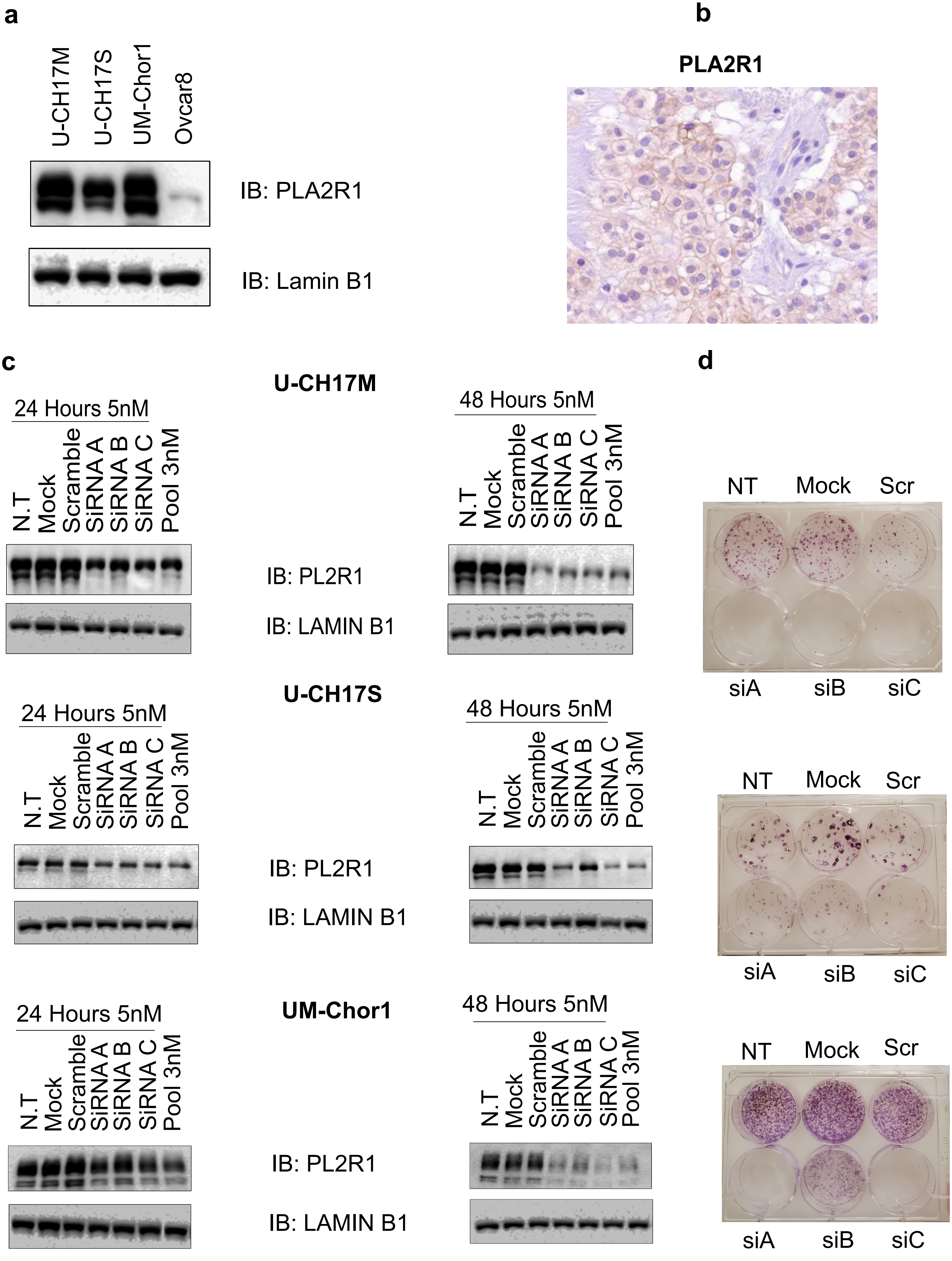

Supplementary Fig. S5

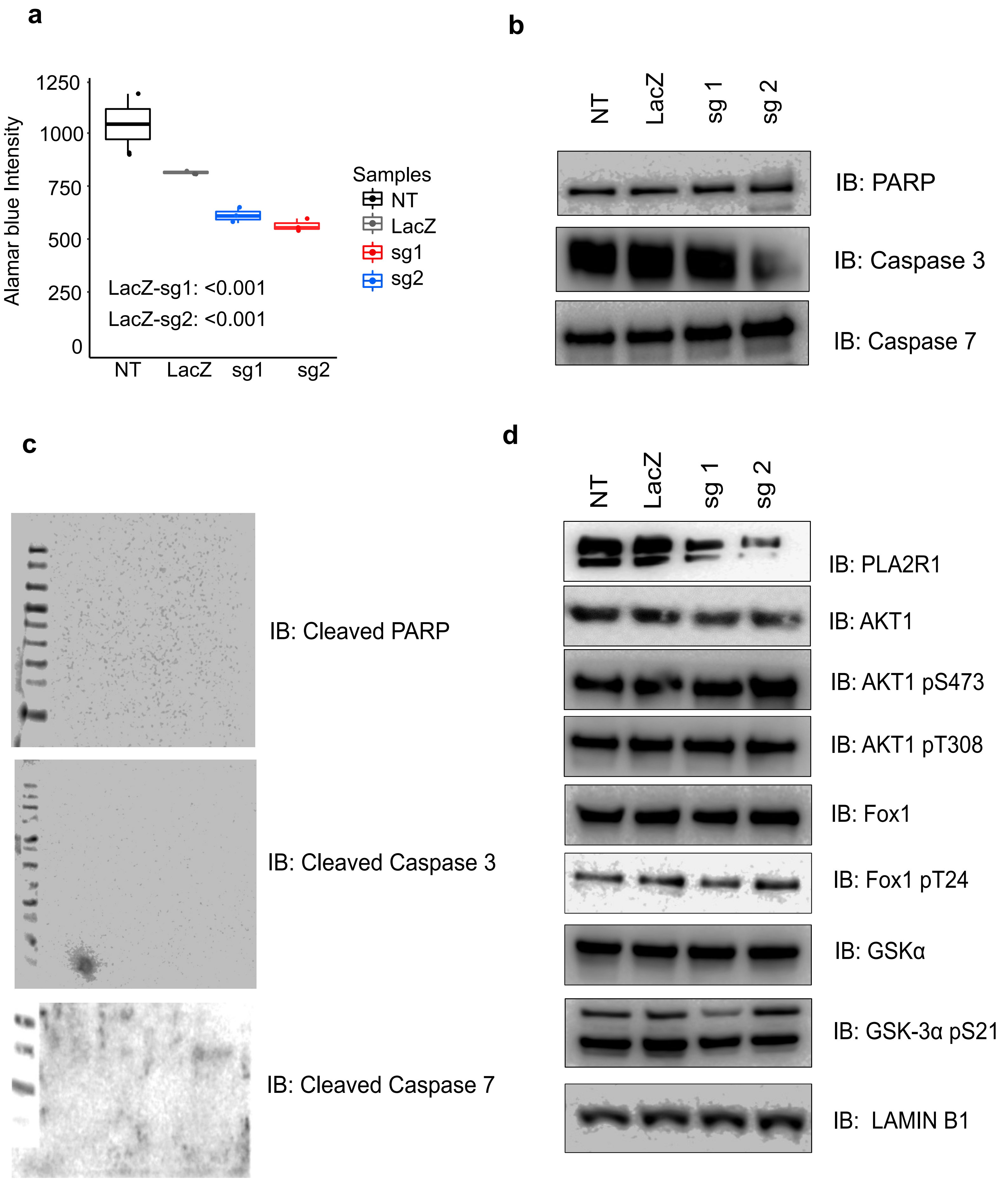

Supplementary Fig. S6

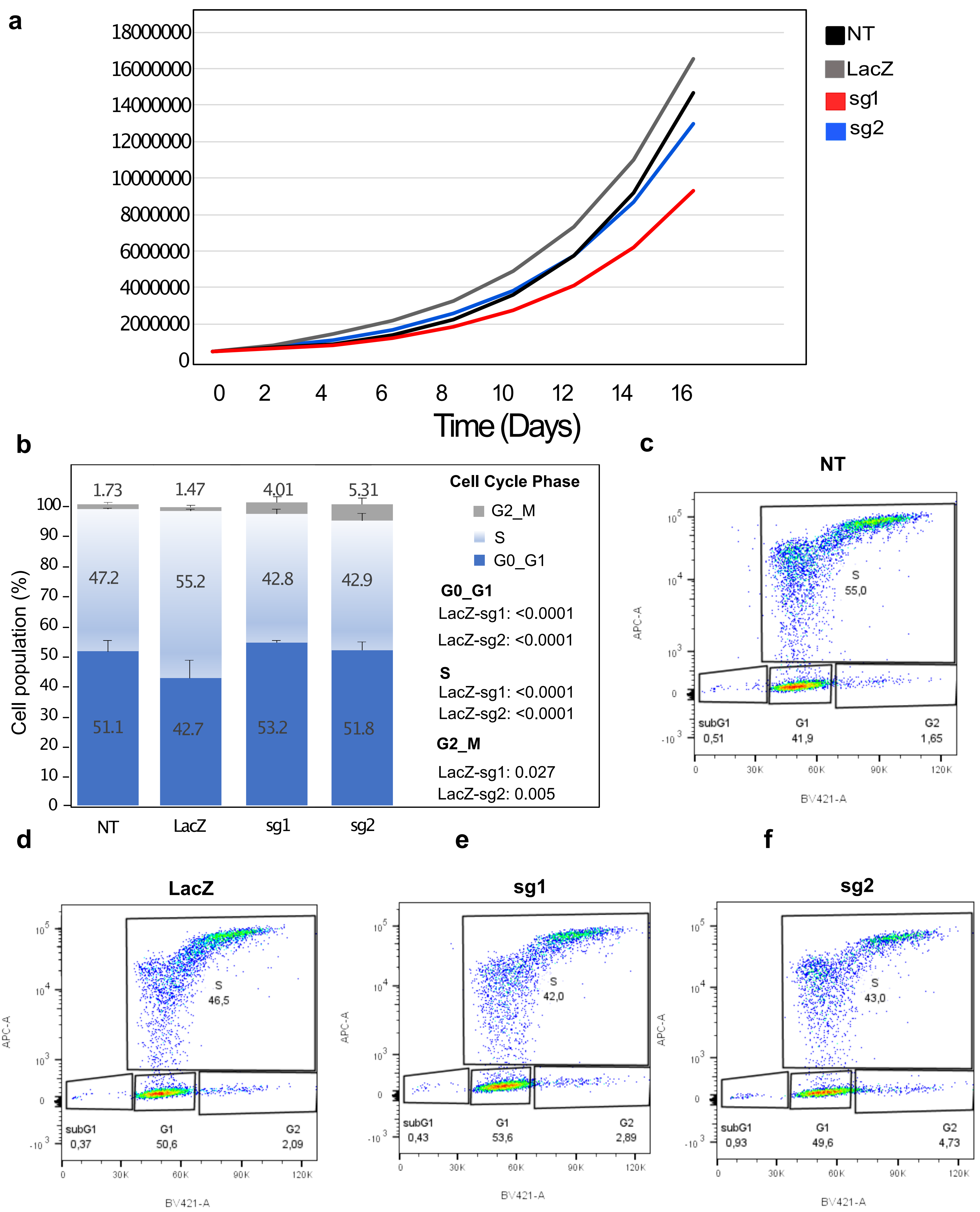

Supplement: Supplementary file 1 — Additional file 1. Fig. S1: Protein detection and subtractive proteomics. a) Bar plot showing the number of proteins detected in each organelle fraction with the dots on the bar representing number of replicates. b) Principal component analysis (PCA) showing proteomic separation among the organelle fractions. c-f) Bar plot showing the number of proteins detected in the four clusters (through hierarchal clustering) corresponding to organelle selective proteins from different databases. Fig. S2: Differential expression analysis between organelle fractions. a) Venn diagram showing overlap between the plasma membrane fraction with all the other organelle fractions in the four chordoma cell lines. Proteins significantly enriched in plasma membrane fractions with a Geniescore of >20 and SPC score of 3 and the following cut-off values (FDR 1) are indicated in red. b) Upset plot showing the overlap of differentially expressed proteins with Genie score and SPC cut-off in the four chordoma cell lines. Fig. S3: Expression of PLA2R1 in HPA and publicly available proteomics and RNA-seq datasets. a) IHC staining of PLA2R1 in normal tissue as reported by HPA. 1 = Low expression; 2 = medium expression; 3 = high expression. b) Bar plot shows log2 iBAQ intensities of PLA2R1 in different normal tissues in the Wang et al. [44] proteomics dataset. c) Boxplots show log2 normalised protein abundance of PLA2R1 in the GTEx proteomics dataset, with bar plot showing number of samples the protein was detected in. D) Boxplots show log2 TPM+1 of PLA2R1 in GTEx transcriptomics dataset, with bar plot showing number of samples the protein was detected in different normal tissues. Fig. S4: Validation of PLA2R1. a) Immunoblots showing expression of PLA2R1 in an additional chordoma cell line, UM-Chor1 and in a negative control ovarian cancer cell line, Ovcar8. b) Representative IHC image showing expression of PLA2R1 localized to cell membrane. c) Expression of PLA2R1 in U-CH17M, U-CH17S and UM-Chor1 c [file 40478_2024_1751_MOESM1_ESM.pdf]
