# Supplementary material for: Organelle resolved proteomics uncovers PLA2R1 as a novel cell surface marker required for chordoma growth
Source: Acta Neuropathol Commun. 2024 Mar 7;12:39. doi: 10.1186/s40478-024-01751-w (PMC10921702; doi:10.1186/s40478-024-01751-w)
Supplement: Supplementary file 4 — Additional file 4. Table S3: Clinical characteristics of IHC patient cohort. [file 40478_2024_1751_MOESM4_ESM.docx]

**Supplementary Table S3:** Clinical characteristics of patient cohort

| **Clinical Characteristics** | | | |
| --- | --- | --- | --- |
|  | | *Mean* | *Range* |
| **Age** | Years | 58 | 33–80 |
|  | | | |
|  | | *Number* | *% in category* |
| **Sex** | Male | 11 | 44.0 |
|  | Female | 14 | 56.0 |
| **Location** | Skull-base | 16 | 64.0 |
|  | Spinal | 9 | 36.0 |
| **Extent of resection** | Gross-total | 9 | 37.5 |
|  | Subtotal | 15 | 62.5 |
| **Adjuvant radiotherapy** | Received | 17 | 68.0 |
|  | Not received | 8 | 32.0 |
