# Supplementary material for: Organelle resolved proteomics uncovers PLA2R1 as a novel cell surface marker required for chordoma growth
Source: Acta Neuropathol Commun. 2024 Mar 7;12:39. doi: 10.1186/s40478-024-01751-w (PMC10921702; doi:10.1186/s40478-024-01751-w)
Supplement: Supplementary file 5 — Additional file 5. Table S4: Univariable and multivariable Cox analysis of IHC data. [file 40478_2024_1751_MOESM5_ESM.docx]

**Supplementary Table S4:** Univariable and multivariable Cox analyses of IHC data

| **Variable** | | **Univariable** | | **Multivariable** | |
| --- | --- | --- | --- | --- | --- |
|  |  | HR (95% CI) | p-value | HR (95% CI) | p-value |
| **Progression-Free Interval** | | | | | |
| PLA2R1 % positivity | Upper tertile vs. lower tertiles | 5.2 (1.5–17.5) | 0.0078 | 7.8 (1.8–32.7) | 0.0052 |
| Extent of resection | STR vs. GTR | 3.1 (0.8–11.5) | 0.0894 | 5.6 (1.3–25.0) | 0.0228 |
| Adjuvant radiotherapy | No vs. Yes | 1.7 (0.6–5.1) | 0.3185 | 3.2 (0.8–12.6) | 0.0921 |
| **Overall Survival** | | | | | |
| PLA2R1 % positivity | Upper tertile vs. lower tertiles | 6.7 (1.7–27.0) | 0.0076 | 11.5 (1.6–80.7) | 0.0144 |
| Extent of resection | STR vs. GTR | 0.9 (0.2–3.7) | 0.8999 | 0.5 (0.1–2.4) | 0.3934 |
| Adjuvant radiotherapy | No vs. Yes | 2.6 (0.7–9.7) | 0.1590 | 0.4 (0.1–2.9) | 0.3933 |
